# Prevalence and factors influencing cognitive impairment among the older adult stroke survivors: a cross-sectional study

**DOI:** 10.3389/fpubh.2023.1254126

**Published:** 2023-09-15

**Authors:** Yanjin Huang, Qi Wang, Ping Zou, Guoping He, Ying Zeng, Jing Yang

**Affiliations:** ^1^School of Nursing, Hengyang Medical School, University of South China, Hengyang, Hunan, China; ^2^Nipissing University, Toronto, ON, Canada; ^3^Xiangya Nursing School, Central South University, Changsha, Hunan, China; ^4^The Second Affiliated Hospital of University of South China, Hengyang, Hunan, China

**Keywords:** stroke, post-stroke, cognitive impairment, potential factors, activity ability

## Abstract

**Background:**

Cognitive impairment as a complication in post-stroke patients has high prevalence throughout the world. However, few studies have focused on the older adult stroke survivors and explored their prevalence and factors of post-stroke cognitive impairment (PSCI). The study aims to evaluate the cognitive status of stroke patients in Hunan Province, China and to determine the potential risk factors associated with PSCI in order to identify the older adult population in advance and promote healthy aging.

**Methods:**

This cross-sectional study was carried out from August to December, 2021. A total of 520 stroke survivors from 6 tertiary hospitals were randomly selected. The information was collected using the general questionnaire, the Barthel Index Rating Scale and the Mini-mental State Examination (MMSE). Analysis was based on descriptive statistics, chi-square test and the significant variables were included in multivariate logistic regression. The reporting of this cross-sectional study followed the STROBE checklist.

**Results:**

A total of 195 older adults (40.37%) were screened for cognitive impairment based on the results of the MMSE score. Patients in the PSCI group had a higher proportion of individuals aged 70 or older (35.90% vs. 24.65%, *p*<0.001). The potential risk factors for post-stroke cognitive impairment in older adults were being aged between 70 and 79 years old (OR = 3.973, 95% CI, 2.346–6.729, *p*<0.001), being aged 80 years or older (OR = 3.590, 95% CI, 1.373–9.387, *p* = 0.009), having a low level of education (OR = 9.183, 95% CI, 5.341–15.789, *p*<0.001), having hypertension (OR = 1.756, 95% CI, 1.121–2.753, *p* = 0.014), and having a dominant hemisphere lesion (OR = 1.880, 95% CI, 1.193–2.962, *p*<0.001).

**Conclusion:**

The prevalence of PSCI was high among Chinese older adults, particularly those aged 80 years or older. The factors identified in our study could assist in the early identification of older adults at risk, develop personalized management plans, and promote healthy aging.

## Introduction

Stroke is one of the main causes of disability and death worldwide (1, 2). According to the American College of Cardiology, more than 100 million people suffer from stroke each year, resulting in over 6.55 million deaths and 143 million DALYs (3). The impact of stroke on the economy, physical health, and psychological wellbeing has been particularly pronounced, resulting in a significant burden of disease (4).About one-third of stroke survivors are likely to experience post-stroke cognitive impairment (PSCI), which is characterized by a decline in cognitive function, including impaired concentration, memory loss, reduced spatial awareness, and decreased perception and executive abilities (5). Furthermore, mild stroke can impact cognition, leading to potential consequences on returning to work, daily living, and overall quality of life (6, 7). A systematic review and meta-analysis found that post-stroke neurocognitive impairment affects 41.4% of stroke survivors in the US, and is more prevalent in other countries, with rates of 58.5% in Australia, 55.3% in Singapore, and 45.1% in India (8). Additionally, it is crucial to note that cognitive function can deteriorate further over time (6). A 5-year follow-up study found that almost half of the individuals who experienced PSCI eventually developed post-stroke dementia (PSD) (9). Without appropriate intervention, cognitive decline can progress to PSD, which is associated with increased mortality risk and reduced median survival time of 5.1 years compared to 8.8 years for stroke survivors without cognitive dysfunction (10). Due to the lack of consensus on the best rehabilitation intervention for PSCI, optimal treatment approaches are yet to be determined (11, 12). Hence, it is of paramount importance to closely pay attention to the changes in cognitive function following stroke and identify individuals with cognitive impairment for personalized management at an early stage.

Over the past few decades, the high-quality development of the global economy and society has resulted in a widespread trend of reduced fertility rates and consistently increasing life expectancies, which has led to the proliferation of deep aging. Currently, developed countries have already entered a phase of super aging, while China are in the early stage of it (13). Studies have shown that there was a strong association between age and the prevalence of cognitive impairment among stroke survivors, with a progressive increase observed in individuals aged 65–85 years starting from the fifth year post-stroke (14). Although amounts of researches have been dedicated to cognitive impairment after stroke in many industrialized countries, little has been done targeting the older adult population (15). To our knowledge, China has the largest older adult base in the world, and it’s estimated that the proportion of people aged 60 and above will rise from 12.4% in 2010 to 28.0% in 2040 (16). The aim of this study was to evaluate the cognitive status of stroke patients in Hunan Province, China and to determine the potential risk factors associated with PSCI in order to identify the older adult population in advance and promote healthy aging.

## Methods

### Study design and participants

A cross-sectional study was conducted in urban communities located in Hunan Province, China from August to December, 2021. The STROBE checklist was used to report the cross-sectional study.

By the end of 2020, the urban population in Changsha City was 8.3 million and in Hengyang City, it was 3.6064 million. The age-standardized incidence rate (per 100,000) of stroke in China was 200 in 2019 (17). Based on the known research scale and a significance level of 0.05, with an allowable error value of ±5%, we calculated that approximately 53,162 stroke patients existed in Changsha and Hengyang. By referring to specific statistical tables and using the corresponding sample size formula, we determined that a sample size of 397 was required. In view of some factors such as sample loss or non-cooperation, we increased the sample size by 30% to reduce potential errors. Ultimately, we determined the final sample size to be 520.

There are totally 16 tertiary hospitals in Changsha and 5 tertiary hospitals in Hengyang, Hunan Province, China. All of these hospitals are general hospitals with numerous departments and ample bed capacity for providing comprehensive medical services. Each hospital was numbered from 01 to 21, and we selected six tertiary hospitals using the cluster sampling method and computer-generated random numbers. The selection was made to meet the required sample size for our study. We obtained patients’ medical information from the medical records department of these six hospitals. Patients who were diagnosed with first-time ischemic stroke and at least 3 months, aged 60 years and above, resided in the urban areas of Changsha or Hengyang communities, with no difficulty in reading comprehension and provided the written informed consent were eligible for the study. According to the Chinese guidelines for the diagnosis and treatment of acute ischemic stroke 2018, the diagnostic criteria for ischemic stroke were the presence of neuroimaging ischemic lesions, or signs or symptoms lasting for more than 24 h (18). Exclusion criteria for patients included a history of cognitive impairment or dementia before the stroke event which was diagnosed by the test of neuropsychology, biomarker assessments, self-report from patients and reports from individuals familiar with the patient (19). Patients suffering from malignant diseases was also be excluded.

### Data collection

Initially, we contacted eligible patients by phone according to their medical records, explaining the study’s purpose and methods to them, and ensuring that their participation was voluntary. Following this, a face-to-face survey was conducted at the Community Health Service Center, where participants were required to sign an informed consent form and questionnaires were distributed by uniformly trained researchers. The researchers would explain any questions the participants may have during the completion process. Any questions raised by the participants during the completion process were explained by the researchers. Once the questionnaires were completed, the researchers checked them for completeness and accuracy of medical information on spot. This study was approved by the Ethics Committee of University of South China (no.4304083006772).

### Study instrument

The general questionnaire was used to collect demographic and medical information of the research subjects. The demographic data included age (60–69 years, 70–79 years, or ≥ 80 years), gender (male or female), pre-retirement occupation (physical labor or mental labor), and marital status (married or other). Educational background and economic income were also self-reported by the participants. The educational background was divided into two levels according to the education system in China: low education experience included education at the elementary school level or below (≤6 years), and high education experience included education at the secondary school level or above (>6 years). According to the Hunan Provincial Bureau of Statistics, the average monthly income of employees was $1055.8 in 2021, which was used as the threshold value for economic income in our study. In terms of lifestyle, the patient’s participation in rehabilitation training after discharge was categorized as either “yes” or “no.” Smoking and drinking status were classified as “yes,” “used to be,” or “no. Regarding medical information, we collected diabetes (yes or no), hypertension (yes or no), time after stroke (3–6 months, or 6–12 months or >12 months), lesion location (non-dominant hemisphere or dominant hemisphere) based on the medical records.

The activity ability of individuals was assessed using the Barthel Index Rating Scale, which was developed by American scholars Dorothy Barthel and Florence Mahney in 1965. This scale measures an individual’s ability to perform activities of daily living and assigns scores ranging from 0 to 100 points. Scores between 0 and 20 are considered severe, while scores between 21 and 60 are classified as moderate. Scores between 61 and 90 indicate a mild disability, while scores between 91 and 100 indicate no disability at all.^25^ In our study, we grouped the scores into two categories: no disability (91–100) or limited disability (≤90).

The Mini-Mental State Examination (MMSE) was developed by Folstein et al. in 1975 and is widely used as a screening tool for cognitive impairment. The Chinese version of MMSE was revised by Mingyuan Zhang et al. and we have obtained the permission for using it. It consists of seven dimensions, including temporal and place orientation, verbal immediate memory, attention and computation, delayed memory, naming, visual space, verbal repetition, comprehension, and expression. Each item is scored on a scale of 0 to 1 point, and the total score ranges from 0 to 30 points. This test can be completed in 5–10 min. Cognitive impairment is indicated when the total score is ≤20 for individuals with low education level (≤6 years), and ≤ 24 for those with high education level (>6 years), according to the Shanghai Mental Health Center (20). The MMSE is a quick and reliable tool with a high Youden index, and a balanced trade-off between sensitivity and specificity (21).

### Data analyzes

Data were analyzed using SPSS 25.0 statistical software. Frequency and percentage were used to describe the categorical variables of post-stroke patients’ general and medical characteristics. We also utilized descriptive statistics to explore the frequency of cognitive impairment among different groups, and the chi-square test was applied to analyze whether there were significant differences in the groups of categorical variables including age, gender, pre-retirement occupation, marital status, educational background, economic income, rehabilitation training, activity ability, smoking status, drinking status, disease history about diabetes and hypertension, time after stroke and lesion location. Furthermore, to identify the potential risk factors associated with post-stroke cognitive impairment, the multivariate logistic regression including the significant variables from the univariate analysis was performed. We computed odds ratios (ORs) and 95% confidence intervals to estimate the level of association between variables. Statistical significance was set at *p* < 0.05.

## Results

### Baseline characteristics of research subjects

We distributed 520 questionnaires, and 483 questionnaires were collected after excluding 37 invalid questionnaires with missing items. The collection efficiency was 92.88%. The demographic and medical characteristics of the sample were outlined in Table 1. In the total sample, there were 312 subjects, with the highest number in the 60–69 years age group (64.60%), followed by 141 subjects aged 70–79 years (29.19%), and 30 subjects in the age group of ≥80 years (6.21%). Females accounted for 51.97% of participants, while males accounted for 48.03%. Approximately 47.62% of patients had an elementary school education or below. The majority of patients were married (78.47%), and 64.60% of patients had engaged in physical labor before retirement. Additionally, 62.94% of patients had a household monthly income higher than the Hunan province mean wage standard ($1055.8). Furthermore, 128 patients (26.50%) reported smoking behavior, while 190 patients (39.34%) reported drinking behavior. The proportions of participants with limited activity ability, rehabilitation training experience, diabetes, or hypertension were 74.95, 40.99, 32.30, and 59.63%, respectively. In terms of stroke information, the majority of patients were 6–12 months post-stroke (54.66%), and 280 patients (57.97%) had lesions located in the non-dominant hemisphere.

**Table 1:** Demographic and medical characteristics of older adult stroke survivors.

Variable	Frequency (*N*)	Percentage (%)
Age		
60–69 years	312	64.60
70–79 years	141	29.19
≥80 years	30	6.21
Gender		
Female	251	51.97
Male	232	48.03
Educational background		
Low education experience	230	47.62
High education experience	253	52.38
Marital status		
Married	379	78.47
Other	104	21.53
Pre-retirement occupation		
Physical labor	312	64.60
Mental labor	171	35.40
Economic income		
<$1055.8 per month	179	37.06
≥$1055.8 per month	304	62.94
Activity ability		
Not limited	121	25.05
Limited	362	74.95
Rehabilitation training		
No	285	59.01
Yes	198	40.99
Smoking		
No	355	73.50
Yes	128	26.50
Drinking		
No	293	60.66
Yes	190	39.34
Diabetes		
No	327	67.70
Yes	156	32.30
Hypertension		
No	195	40.47
Yes	288	59.63
Time after stroke		
3–6 months	142	29.40
6–12 months	264	54.66
>12 months	77	15.94
Lesion location		
Non-dominant hemisphere	280	57.97
Dominant hemisphere	203	42.03

### Prevalence of cognitive impairment among older adult stroke survivors

A total of 195 older adults (40.37%) were screened for cognitive impairment based on the results of the MMSE score (Table 2). Patients in the PSCI group had a higher proportion of individuals aged 70 or older (35.90% vs. 24.65%, *p*<0.001), with low education experience (75.38% vs. 28.82%, *p*<0.001) and hypertension (67.18% vs. 54.51%, *p* = 0.005), and their lesion location were more likely to be dominant hemisphere (48.72% vs. 37.50%, *p* = 0.014) compared to the Non-PSCI group. The proportion of Non-PSCI group patients who were engaged in physical labor before retiring is higher compared to the PSCI group patients (74.31% vs. 50.26%, *p*<0.001). The PSCI group of patients had a slightly lower frequency of limited activity ability than the Non-PSCI group (68.21% vs. 79.51%, *p* = 0.005). However, the number of individuals with activity ability limitations is more than twice as much as the unlimited population (68.21% vs. 31.79%) in the PSCI group.

**Table 2:** Chi-square test for differences in demographic and medical characteristics between groups.

Variable	Non-PSCI (*N* = 288)	PSCI (*N* = 195)	Prevalence (%)	*p*-value
Age				
60–69 years	209 (72.57)	103(52.82)	33.01	<0.001
70–79 years	71 (24.65)	70 (35.90)	49.65	
≥80 years	8 (2.78)	22 (11.28)	73.33	
Gender				
Female	151 (52.43)	100 (51.28)	39.84	0.804
Male	137 (47.60)	95 (48.72)	40.95	
Educational background				
Low education experience	83 (28.82)	147 (75.38)	63.91	<0.001
High education experience	205 (71.18)	48 (24.62)	18.97	
Marital status				
Married	223 (77.43)	156 (80.00)	41.16	0.500
Other	65 (22.57)	39 (20.00)	37.50	
Pre-retirement occupation				
Physical labor	214 (74.31)	98 (50.26)	31.41	<0.001
Mental labor	74 (25.69)	97 (49.74)	56.73	
Economic income
<$1055.8 per month	104 (36.11)	75 (38.46)	41.90	0.600
≥$1055.8 per month	184 (63.89)	120 (61.54)	39.47	
Activity ability				
Not limited	59 (20.49)	62 (31.79)	51.24	0.005
Limited	229 (79.51)	133 (68.21)	36.74	
Rehabilitation training				
No	163 (56.60)	122 (62.56)	42.81	0.191
Yes	125 (43.40)	73 (37.44)	36.87	
Smoking				
No	207 (71.88)	148 (75.90)	41.69	0.326
Yes	81 (28.13)	47 (24.10)	36.72	
Drinking				
No	177 (61.46)	116 (59.49)	39.60	0.663
Yes	111 (38.54)	79 (40.51)	41.58	
Diabetes				
No	191 (66.32)	136 (69.74)	41.59	0.430
Yes	97 (33.68)	59 (30.26)	37.82	
Hypertension				
No	131 (45.49)	64 (32.82)	32.82	0.005
Yes	157 (54.51)	131 (67.18)	45.49	
Time after stroke				
3–6 months	86 (29.86)	56 (28.72)	39.44	0.952
6–12 months	157 (54.51)	107 (54.87)	40.53	
>12 months	45 (15.63)	32 (16.41)	41.56	
Lesion location				
Non-dominant hemisphere	180 (62.50)	100 (51.28)	35.71	0.014
Dominant hemisphere	108 (37.50)	95 (48.72)	46.80	

**Figure 1:**
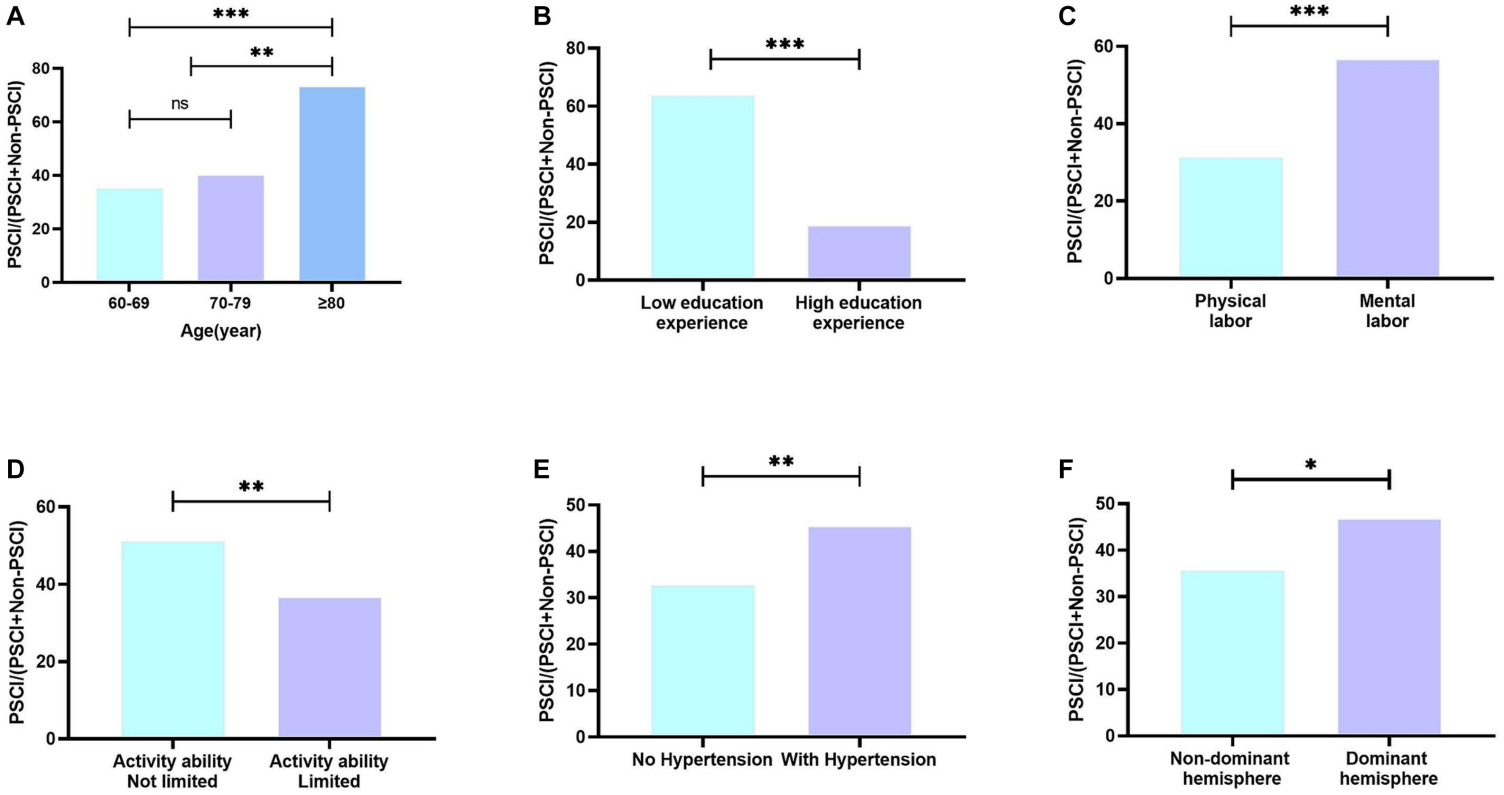
Prevalence of PSCI in different groups. **(A)** Prevalence of PSCI in different age groups; **(B)** Prevalence of PSCI in different education background groups; **(C)** Prevalence of PSCI in different pre-retirement occupation groups; **(D)** Prevalence of PSCI in different activity ability groups; **(E)** Prevalence of PSCI in different hypertension situation groups; **(F)** Prevalence of PSCI in different lesion location; ns means no significance; *p < 0.05; **p < 0.01; ***p < 0.001.

As shown in Figure 1, the prevalence of PSCI increased with age, patients aged 80 or older had the highest incidence reaching 73.33%. The prevalence decreased as the number of years of schooling increased (18.97% vs. 63.91%, *p*<0.001). Patients who engaged in mental labor before retirement had a higher prevalence compared to those engaged in physical labor (56.73% vs. 31.41%, *p*<0.001). Furthermore, a significant difference in prevalence was observed based on activity ability (51.24% vs. 36.74%, *p*<0.01). Regarding medical characteristics, patients with hypertension had a higher prevalence compared to those without hypertension (45.49% vs. 32.82%, *p*<0.01). The prevalence of PSCI in the dominant hemisphere was significantly higher than that in the non-dominant hemisphere (46.80% vs. 35.71%, *p*<0.05).

### Factors of older adult post-stroke cognitive impairment patients

**Figure 2:**
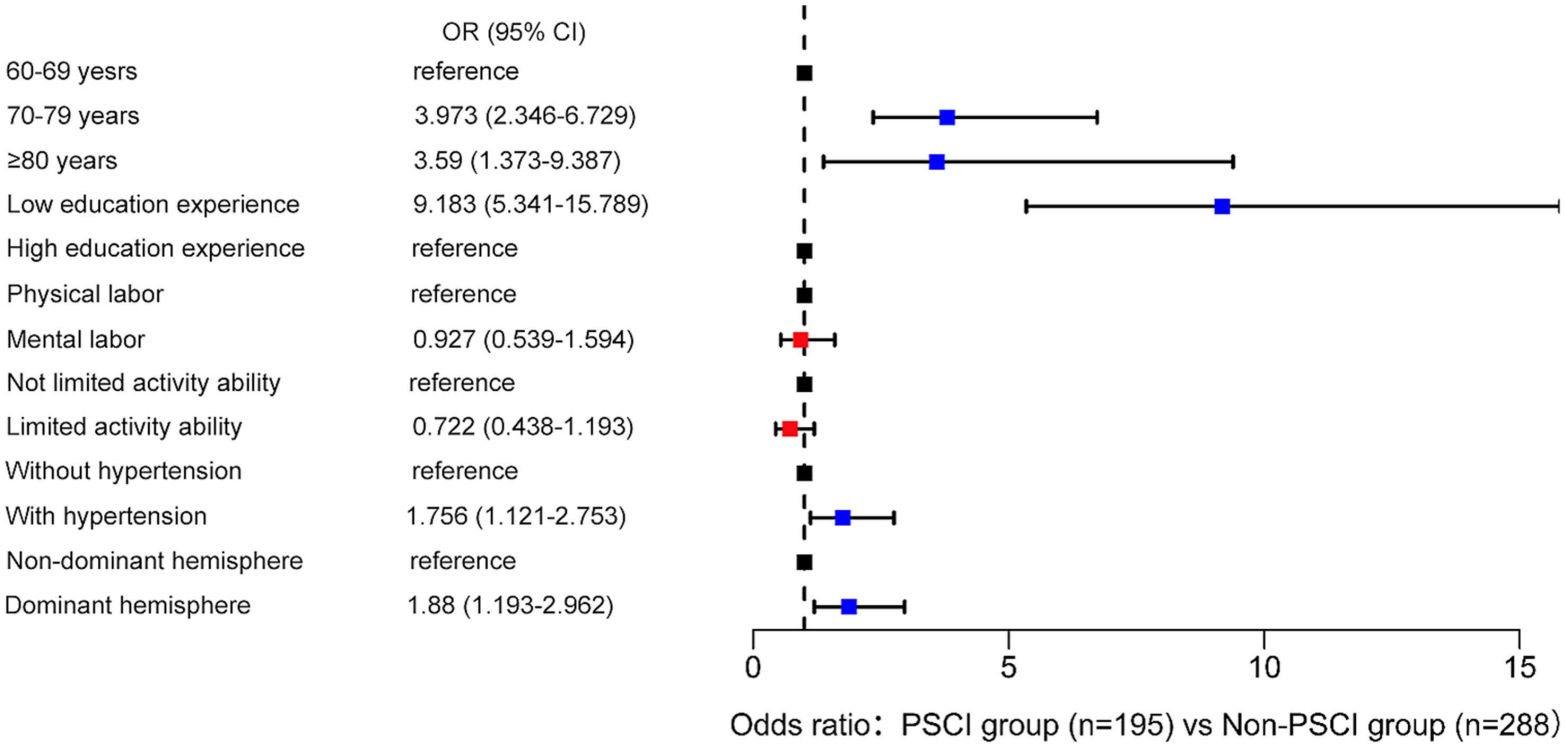
Association of demographic and medical information with PSCI group compared with Non-PSCI group.

Differences in demographic and medical characteristics between PSCI group and Non-PSCI group were reported in Table 2. Age, educational background, pre-retirement occupation, activity ability, hypertension status and lesion location were found to be significantly associated with PSCI in older adult stroke survivors based on the univariate analysis (*p*<0.05). These variables were included in multivariate logistic regression analysis to explore risk factors for influencing PSCI in older adults, with the Non-PSCI group as the reference. Association of demographic and medical information with PSCI group compared to the Non-PSCI group was shown in Figure 2. Table 3 showed that patients aged 70 years old or older were more likely to have PSCI compared to patients of 60–69 years old (OR = 3.973, *p*<0.001; OR = 3.590, *p* = 0.009). Low education experience may be a risk factor with PSCI in the older adult population (OR = 9.183, *p*<0.001). Patients with hypertension (OR = 1.75, *p* = 0.014) and those with dominant hemisphere lesions (OR = 1.880, *p*<0.001) were also at increased risk for suffering from PSCI compared with older adults without hypertension and with non-dominant hemisphere lesions. There was no significant association between pre-retirement occupation and activity ability with the occurrence of PSCI.

**Table 3:** Multivariate logistic regression analyzes for potential predictors of PSCI in the older adult population.

Variable	OR	95%CI	*P*-value
Age			
60–69 years	Ref		
70–79 years	3.973	2.346–6.729	<0.001
≥80 years	3.59	1.373–9.387	0.009
Educational background			
Low education experience	9.183	5.341–15.789	<0.001
High education experience	Ref		
Pre-retirement occupation			
Physical labor	Ref		
Mental labor	0.927	0.539–1.594	0.784
Activity ability			
Not limited	Ref		
Limited	0.722	0.438–1.193	0.204
Hypertension			
No	Ref		
Yes	1.756	1.121–2.753	0.014
Lesion location			
Non-dominant hemisphere	Ref		
Dominant hemisphere	1.88	1.193–2.962	<0.001

## Discussion

This cross-sectional study investigated the prevalence of PSCI and potential risk factors of cognitive impairment among older adult stroke survivors in Hunan Province, China. The study estimated a prevalence rate of 40.73% for PSCI in older adult patients. Some characteristics including 70 years old or older, low education experience, hypertension and dominant hemisphere lesions were identified as risk factors for PSCI in older adults.

On a global scale, the occurrence rate of PSCI is considerable. A synthesis of 13 studies conducted by the Stroke and Cognition Consortium (STROKOG) across 8 countries found that 44% of stroke survivors had global cognitive impairment, with 30–35% experiencing domain-specific impairments (22). But the prevalence rates of PSCI vary across different studies, which may be attributed to differences in population characteristics, study settings, assessment timing, cognitive tests used, and cutoff values (23). In our study, we utilized the authorized Chinese version of the MMSE scale, which was adapted to align with the cultural background and characteristics of the Chinese study population. This ensured the precision and accuracy of the measurement results. Our study found a cognitive impairment rate of 40.73% in older adult stroke patients. Comparatively, another study conducted on the older adult population reported a lower prevalence rate of cognitive impairment at 22.24% (24). This difference may be attributed to the focus on the older adult population with stroke, as stroke is a neurological condition that affects blood supply to the brain and can impair cognitive function, thereby increasing the risk of cognitive impairment (5). As this study reported, individuals aged 70 and above was the potential risk factor of PSCI. According to the most recent study conducted in China, individuals aged 75 and above accounted for over 80% of the total PSCI population (25). China’s efforts in older adult healthcare began relatively late, resulting in most individuals undergoing the natural aging process as they grow older, which brought decreased physical capabilities without the involvement of healthcare and maintenance. This age-related decline in physical function and activity has a significant impact on overall health (26). We found that nearly half of the older adult stroke patients exhibited cognitive impairment, although the overall prevalence was not particularly high. This could be attributed to limitations in our sample source, which was restricted to residents of secondary cities’ communities, predominantly in the 60–69 age group. However, we observed a remarkably high occurrence of cognitive impairment among individuals aged 80 and above, reaching 73.33%. The aging population has become a significant national challenge for China in this century. The number of individuals aged 60 and above is projected to exceed 300 million during the “Fourteenth Five-Year Plan” period, officially marking the transition into the stage of moderate aging. In the context of rapid growth in the older adult population, the size of the older adult stroke population is also expanding (24). Given that cognitive impairment is a significant complication of stroke, there is a pressing need for more research on cognitive impairment in older adults. It is crucial to identify individuals at risk for PSCI as early as possible in order to promote active aging.

This study also observed differences in the prevalence of PSCI among individuals with different educational background, occupational nature before retirement, activity ability, blood pressure, and lesion locations which are consistent with other studies in various countries (27–29). Specifically, when discussing the prevalence of PSCI under different conditions, this study found that the proportion of stroke patients who engaged in mental labor before retirement who suffer from cognitive impairment was actually higher than that of those involved in physical labor, which differs from previous research (25). The number of participants engaged in mental labor in this study was relatively small, approximately half of the number of participants engaged in physical labor. This may be a contributing factor to the higher prevalence of PSCI compared to physical workers. Regular activity is crucial for protecting and improving cognitive function (30). However, the proportion of older adult without limited activity ability who develop PSCI was higher than that of those with physical disability in our study. In this study, as it focused on older adults, approximately 75% of the sample had varying degrees of limited activity ability. Consequently, the population of individuals with good physical function was relatively small, resulting in a higher proportion of stroke patients without limited activity ability in terms of PSCI incidence. On the other hand, within the PSCI population of this study, around 70% had dysfunction in their activity ability, indicating a generally poor physical function among PSCI individuals.

This study also demonstrated that different educational background were associated with the cognitive function in older adult stroke patients. Cognitive function refers to the ability to utilize and interpret knowledge for problem-solving, and it is positively associated with literacy. Individuals with higher educational background are more likely to possess scientific literacy, allowing them to acquire knowledge about their condition and utilize relevant health education resources. Conversely, survivors with lower education level tend to have poor understanding of disease-related knowledge and low compliance. They usually neglect their changes in symptoms and function, failing to intervene in the progression of complications, ultimately leading to more severe health outcomes (31). This may explain why individuals with lower level of education are more prone to experiencing impairment in cognitive function. In addition, hypertension is also the factor of PSCI according to our study. Generally, hypertensive patients tend to have poor self-management ability, scoring low in areas such as diet, exercise, lifestyle habits, and management of risk factors (32). This may contribute to a certain degree of harm to patients’ overall health. At the same time, hypertension is a kind of cerebrovascular disease which is one of the main causes of cognitive impairment. It will impair the structural integrity and network function, promote neurovascular dysfunction, blood–brain barrier disruption, lacunar infarcts, and white matter damage, all of which contribute to the exacerbation of cognitive decline. Studies have shown that hypertension is the most common risk factor for cognitive impairment and significantly increases the risk of cognitive impairment in older adults and exacerbates the development of Alzheimer’s disease (33). Gottesman et al. (34) conducted a 20-year follow-up study on 13,476 hypertensive patients and found a significant association between elevated systolic blood pressure and late-life cognitive decline. Recent prospective studies have also found that compared to individuals with normal midlife to late-life blood pressure, those with persistent hypertension have an increased risk of dementia (35). The lesion location is also an important factor of PSCI in this study which is consistent with other studies (36, 37). The dominant hemisphere is typically responsible for a range of cognitive functions, including language, spatial cognition, memory, and executive functioning. When a cerebrovascular accident, especially a stroke, occurs and affects the dominant hemisphere, it can lead to cognitive impairments. However, the specific infarct locations have not been clearly defined yet. Zhao et al. (38). suggested that left cerebral hemisphere, left basal ganglia, left frontal lobe, left thalamus, and PSCI have been associated significantly.

There are some limitations that should be noted. First, this study made use of a cross-sectional design which cannot determine the casual relationship between variables. It can only analyze the potential predicators of PSCI. Second, the study population was limited to Hunan province, and future research should conduct nationwide multicenter investigations specifically targeting older adult stroke patients. Third, the baseline stroke severity was not assessed due to the absence of NIHSS measurements. Forth, there were insufficient variables recorded in our study. We will combine with more measurable variables in the medical field for analysis in future studies. Additionally, the specific types and duration of rehabilitation training were not clearly defined in our study. The heterogeneity of the population might have grouped individuals into the same category, which could be a reason why the study did not find any impact of rehabilitative exercises on cognitive function.

## Conclusion

The prevalence of post-stroke cognitive impairment in older adult patients was 40.73% in this cross-sectional study, and it varied among age, educational background, pre-retirement nature, activity ability, hypertension and lesion location. The results also revealed that the potential risk factors of cognitive impairment were advanced age (≥70 years old), low educational level, hypertension, and dominant hemisphere lesion. We hope these characteristics could help identify people at risk as early as possible and tailored intervention should be performed for them.

## Data availability statement

The raw data supporting the conclusions of this article will be made available by the authors, without undue reservation.

## Ethics statement

The studies involving humans were approved by Ethics Committee of University of South China. The studies were conducted in accordance with the local legislation and institutional requirements. The participants provided their written informed consent to participate in this study.

## Author contributions

YH: Conceptualization, Formal Analysis, Funding acquisition, Methodology, Software, Writing – original draft. QW: Formal Analysis, Investigation, Software, Writing – original draft. PZ: Writing – review & editing. GH: Supervision, Writing – review & editing. YZ: Supervision, Writing – review & editing. JY: Conceptualization, Funding acquisition, Investigation, Supervision, Writing – review & editing.

## Funding

This study was supported by the Excellent Youth Foundation of Educational Commission of Hunan Province, China [grant number: 20b495], the Guidance Projects of Science and Technology Department of Hengyang, Hunan Province, China [grant number: s2018F9C31022244 and 2019124] as well as the Research Project of Hunan Provincial Health Commission [grant number: B202314057822].

## Conflict of interest

The authors declare that the research was conducted in the absence of any commercial or financial relationships that could be construed as a potential conflict of interest.

## Publisher’s note

All claims expressed in this article are solely those of the authors and do not necessarily represent those of their affiliated organizations, or those of the publisher, the editors and the reviewers. Any product that may be evaluated in this article, or claim that may be made by its manufacturer, is not guaranteed or endorsed by the publisher.
